# Quantitative pupillometry as a sensitive tool for detecting hydrocephalus-related physiologic burden after aSAH: a prospective feasibility study

**DOI:** 10.1007/s10072-026-08928-2

**Published:** 2026-03-03

**Authors:** Jonas Lin, Dorothea Mitschang, Viktoriya Sydorenko, Alexander Younsi, Ahmed El Damaty, Martin Dugas, Sandro M. Krieg, Pavlina Lenga

**Affiliations:** 1 Department of Neurosurgery, Heidelberg University Hospital, University of Heidelberg, Im Neuenheimer Feld 400, Heidelberg, 69120 Germany; 2 Medical Faculty of Heidelberg University, Heidelberg, Germany; 3 Department of Neurosurgery, BG Klinik Ludwigshafen, Ludwigshafen, Germany; 4 Institute of Medical Informatics, Heidelberg University Hospital, Heidelberg, Germany

**Keywords:** Subarachnoid hemorrhage, Hydrocephalus, Quantitative pupillometry, Ventriculoperitoneal shunt, Neurological pupil index

## Abstract

**Background:**

Hydrocephalus after aneurysmal subarachnoid hemorrhage (aSAH) is common, but bedside markers of its physiological burden are limited. Quantitative pupillometry (QP) is objective and fast; whether it mirrors hydrocephalus severity is uncertain.

**Methods:**

Single-center cohort of adults with aSAH undergoing ventriculoperitoneal shunting (*n* = 17). Automated infrared pupillometry (NPi-200) was performed the day before surgery and on post-op day 1, recording Neurological Pupil index (NPi) and constriction/dilation velocities (CV/DV) as overall values (mean of both eyes) and inter-eye asymmetry (|OD–OS|). Pre-op CT markers were combined into a standardized Hydrocephalus Severity Score (HSS; higher=worse).

**Results:**

Mean pre-op NPi was 3.81 ± 0.69 and post-op 4.03 ± 0.66; median ΔNPi = + 0.13 (Wilcoxon *p* = 0.141). Responders (ΔNPi ≥ + 0.5) were 5/14 (35.7%). Cross-sectionally, overall values did not mirror ventricular size: NPi vs. HSS (ρ = 0.37, *p* = 0.141, q = 0.423) and NPi vs. Evans index (EI) (ρ = 0.09, *p* = 0.735, q = 0.735) were non-significant; CV/DV vs. HSS were likewise neutral. In contrast, inter-eye CV asymmetry correlated with HSS (ρ = 0.68, *p* = 0.006, q = 0.017; *n* = 16) and trended with Evans (ρ = 0.49, *p* = 0.054, q = 0.161). In age/sex-adjusted models, no radiologic metric, including HSS, reliably explained pathological NPi (< 3).

**Conclusions:**

In shunt-dependent aSAH hydrocephalus, pattern matters more than absolute value. Absolute NPi and mean velocities did not reflect ventricular size, whereas inter-eye constriction-velocity asymmetry captured composite radiologic burden, consistent with lateralized parasympathetic stress. Around shunt placement, QP behaved as a dynamic biomarker, revealing patient-level gains despite flat cohort averages. Findings support trend- and asymmetry-based pupillometry to complement imaging for CSF-pathway reassessment and early post-shunt surveillance, warranting prospective validation.

**Supplementary Information:**

The online version contains supplementary material available at 10.1007/s10072-026-08928-2.

## Introduction

Hydrocephalus is a common and potentially devastating complication of aneurysmal subarachnoid hemorrhage (aSAH), reported in roughly 20–30% of cases and arising acutely or in delayed fashion as malresorptive cerebrospinal-fluid (CSF) failure. Early recognition matters: unchecked ventricular enlargement and raised intracranial pressure (ICP) can precipitate brainstem compromise, whereas timely CSF diversion—often external ventricular drainage (EVD) followed by ventriculoperitoneal (VP) shunting in non-resolving cases—improves cerebral perfusion and outcomes. Radiographically, ventricular size is commonly tracked with reproducible linear indices on CT that correlate with ventriculomegaly severity and are practical at the bedside [1–3]. Clinically, the pupillary light reflex (PLR) is among the most sensitive bedside signs of rising ICP, but traditional manual exams suffer from inter-rater variability and subjective descriptors. Automated infrared pupillometry mitigates this by quantifying pupil size and dynamics and reporting a composite Neurological Pupil index (NPi) from 0 to 5, where values ≥ 3 are considered within normal limits [4–6].

Growing neuro-ICU evidence body suggests quantitative pupillometry behaves as a “neurologic vital sign.” In case series and observational cohorts across acute brain injury, abnormal or falling NPi often precedes clinically overt transtentorial herniation and other neurological downturns; in one hospital-onset unresponsiveness cohort, an NPi cut-off < 1.6 identified herniation with high specificity. Moreover, in invasively monitored patients, sustained ICP elevations are associated with concomitant NPi depression that improves after hyperosmolar therapy, underscoring the physiological coupling between PLR dynamics and intracranial compliance [7].

Whether these pupillometric signatures also capture the *subtle* ventricular distension of post-aSAH hydrocephalus is unsettled. In a aSAH cohort, Campos et al. found no significant correlation between NPi values and a CT-derived hydrocephalus severity score despite frequent ventriculomegaly, highlighting a potential insensitivity of *mean* NPi to mild–moderate ventricular enlargement [8]. Conversely, during EVD clamp trials, an NPi < 3 showed perfect specificity for early clamp-failure—i.e., inability to tolerate CSF outflow occlusion—suggesting that *dynamic* deteriorations in pupillary reactivity may herald clinically significant (mal)resorptive hydrocephalus even when static imaging changes are modest [9]. Large multicentre data further support NPi as a meaningful physiologic biomarker after acute brain injury, while reinforcing that it complements rather than replaces invasive ICP monitoring [10].

Accordingly, we conducted a single-center feasibility study (*n* = 17) to test whether quantitative pupillometry—particularly the NPi and related pupillary light-reflex metrics—can detect the presence of, and resolution after, hydrocephalus in patients with aneurysmal subarachnoid hemorrhage undergoing VP shunt placement. Pairing standardized pupillometry with reproducible CT-based ventricular indices, we evaluate whether NPi provides an early, objective marker of subtle hydrocephalus after aSAH and a practical adjunct for shunt decision-making.

## Methods

### Study design and population

We conducted a single-center observational cohort of 17 consecutive adults with aSAH who underwent VP shunting for malresorptive hydrocephalus at our neurosurgical department. Eligibility required a confirmed aSAH, a preoperative cranial CT (cCT) suitable for quantitative ventricle assessment, and standardized QP within predefined windows. We excluded eyes/assessments with conditions likely to invalidate QP at the time of testing (e.g., fixed surgical mydriasis, dense corneal opacity, acute third-nerve palsy unrelated to hydrocephalus) or deep neuromuscular blockade; previous cranial procedures and external ventricular drainage were not exclusionary. The protocol complied with the Declaration of Helsinki and received approval from the local ethics committee (approval number S518/2023); data handling followed institutional GDPR policies.

### Pupillometry

Automated infrared pupillometry (NPi-200, Neurooptics) was performed at the bedside under standardized light conditions before and after shunt placement, assessing pupil size, latency, and constriction/dilation velocities. These parameters provide objective assessment of afferent and efferent pupillary reflex pathways. The resulting NPi is an algorithm-generated score ranging from 0 to 5; values between 3 and 5 are considered normal, whereas values below 3 indicate an abnormal pupillary light reflex suggestive of neurological dysfunction [11]. The NPi was measured for each eye separately on the day before surgery and on postoperative day 1 to capture acute changes associated with shunt implantation. Because automated pupillometry exhibits low inter-observer variability [12], we did not repeat measurements at the same time point. Responders were defined a priori as patients with an increase in overall NPi of **ΔNPi ≥ 0.5**, chosen to represent a within-patient change that exceeds expected short-interval measurement/device variability reported for automated pupillometry [1].

### Clinical and radiological data

We captured demographics, GCS at the time of pre-op QP, and symptoms (headache, nausea/vomiting). Primary endpoints were the cross-sectional associations of pre-op overall NPi with EI and HSS. Key secondary endpoints included: (i) pre-op overall CV/DV vs. HSS; (ii) asymmetry (|OD–OS|) of QP metrics vs. HSS/EI; (iii) pre→post change in overall NPi; and (iv) responder rate, prespecified as ΔNPi ≥ + 0.5. A “rescue” response was predefined for patients with pre-op NPi < 3 achieving post-op NPi ≥ 3 Preoperative cCT (admission/pre-op) was reviewed on PACS by two experienced neurosurgeons (J.L., P. L.) blinded to QP. We extracted standardized indices of ventriculomegaly: Evans index (EI), bicaudate index (BCI), third-ventricle width (TVW), temporal-horn diameter (THD), callosal angle (CA), and periventricular lucency (PVL; present/absent) (measurement protocol in Supplementary Methods). Ventricular volume (cm³) was segmented in 3D (Origin^®^, Brainlab v3.1) with slice-by-slice correction as needed.

### Composite hydrocephalus severity score (HSS)

To summarize radiologic burden and reduce collinearity, we built a patient-level Hydrocephalus Severity Score (HSS) from EI, BCI, ventricular volume, THD, TVW, PVL (1/0) and –CA (negative sign so that smaller angles increase severity). Each component was z-standardized to the cohort mean/SD; the HSS was the mean of available z-scores (requirement ≥ 4/7 components). Higher HSS denotes greater hydrocephalus severity.

### Statistical analysis

Continuous variables are summarized as mean ± SD or median (IQR, range) as appropriate; categorical variables as n (%). For each pupillometry metric (Neurological Pupil index [NPi], size before constriction, constriction velocity [CV], dilation velocity [DV]) we computed an overall value per patient as the row-wise mean of OD and OS and an asymmetry value as the absolute inter-eye difference |OD–OS|. NPi < 3 was prespecified as pathological. To capture radiologic hydrocephalus burden while limiting collinearity, we derived a HSS by orienting all components so that higher values reflected worse severity (Evans index, bicaudate index, third-ventricle volume, temporal-horn diameter, third-ventricle width, periventricular lucency [1/0], and the negative of callosal angle), z-standardizing each component to the cohort mean and SD, and averaging the available z-scores per patient (requirement ≥ 4/7 components). Cross-sectional associations between preoperative pupillometry (overall and asymmetry) and imaging markers/HSS or GCS used Spearman rank correlation (two-sided). Pre- to postoperative changes in overall NPi used the Wilcoxon signed-rank test; the proportion achieving a clinically relevant improvement was reported for ΔNPi ≥ + 0.5 with exact (Clopper–Pearson) 95% CIs. The odds of pathological overall NPi (< 3) at baseline were explored with logistic regression adjusted for age and sex; predictors (HSS and individual imaging metrics, with callosal angle inverted) were scaled per 1 SD to yield per-SD odds ratios with 95% CIs. Multiplicity was controlled with Benjamini–Hochberg false-discovery rate within analysis families (α = 0.05). All statistical analyses were conducted using R (version 4.2.0, R Foundation, Vienna, Austria) and SPSS (version 24.0, IBM Corp., Armonk, NY, USA).

### IRB/IACUC approval

All articles using clinical samples or data and those involving animals must include information on the IRB/IACUC approval or waiver and informed consent. An example is shown below. “We conducted this study in compliance with the principles of the Declaration of Helsinki. The study’s protocol was reviewed and approved by the Institutional Review Board of OO (IRB no. OO). Written informed consent was obtained/Informed consent was waived.”

### Description of participants

Ensure the correct use of the terms “sex” (when reporting biological factors) and “gender” (identity, psychosocial, or cultural factors), and, unless inappropriate, report the sex and/or gender of study participants, the sex of animals or cells, and describe the methods used to determine sex and gender. If the study was done involving an exclusive population, for example, in only one sex, authors should justify why, except in obvious cases (e.g., ovarian cancer). Authors should define how they determined race or ethnicity and justify their relevance.

## Results

### Cohort and baseline

Seventeen patients with aSAH who underwent ventriculoperitoneal shunting were analyzed (mean age 59.9 ± 15.8 years; 10/17 [58.8%] female). At the time of preoperative pupillometry, the Glasgow Coma Scale (GCS) distribution was: 15 in 7/17 (41.2%), 14 in 4/17 (23.5%), 13 in 4/17 (23.5%), 12 in 1/17 (5.9%), and 3 in 1/17 (5.9%). Headache was present in 8/17 (47.1%) and nausea/vomiting in 6/17 (35.3%) (Table 1).

**Table 1:** Baseline characteristics, pre-/postoperative pupillometry, and imaging

Parameter	Value
Mean age (years) (SD)	59.9 (15.8)
Sex (n, %)	
Female	10 (58.8)
Male	7 (41.2)
GCS (mean ± SD)	14.3 (2.3)
Preoperative pupillometry – Overall (mean ± SD)	
NPi	3.8 (0.7)
Constriction velocity (mm/s)	2.4 (1.4)
Dilation velocity (mm/s)	1.0 (0.4)
Preoperative pupillometry – Right (median [IQR, range])	
NPi	3.8 (0.4, 2.1–4.8)
Constriction velocity (mm/s)	2.1 (2.0, 0.7–4.4)
Dilation velocity (mm/s)	1.1 (0.8, 0.2–1.7)
Preoperative pupillometry – Left (median [IQR, range])	
NPi	3.8 (1.0, 2.5–4.8)
Constriction velocity (mm/s)	2.6 (2.0, 0.7–4.9)
Dilation velocity (mm/s)	1.1 (0.7, 0.3–1.6)
Postoperative pupillometry – Overall (mean ± SD)	
NPi	4.0 (0.7)
Constriction velocity (mm/s)	2.5 (1.0)
Dilation velocity (mm/s)	1.0 (0.4)
Imaging (median [IQR, range])	
Evans index	0.3 (0.1, 0.3–0.4)
Third ventricle volume (cm³)	75.4 (37.1, 26.9–239.6)
Callosal angle (°)	88.9 (22.7, 59.8–126.6)
Temporal horn diameter (mm)	80.0 (21.0, 41.0–102.0)
Bicaudate index	0.2 (0.1, 0.2–0.3)
Periventricular lucency (1/0)	1.0 (0.0, 1.0–1.0)
Third ventricle width (mm)	126.0 (20.0, 66.0–184.0)

Data are presented as mean (SD), median [IQR, range], or n (%), as indicated. Quantitative pupillometry was performed using automated infrared pupillometry (NPi‑200, NeurOptics) on the day before ventriculoperitoneal shunt implantation (“Preoperative”) and on postoperative day 1 (“Postoperative”). “Overall” pupillometry values represent the mean of right and left eyes (OD and OS); if one eye was missing/unreliable at a time point, the available eye was used. Imaging markers were measured on the preoperative cranial CT and include Evans index (EI), bicaudate index (BCI), callosal angle (CA, degrees), third‑ventricle width (TVW, mm), temporal‑horn diameter (THD, mm), periventricular lucency (PVL; present=1/absent=0), and ventricular volume (cm³). Measurement definitions are provided in Supplementary Methods. Note: p values are two-sided and unadjusted (nominal). Where referenced in the manuscript, q values denote Benjamini–Hochberg false discovery rate (FDR)-adjusted p values within the corresponding analysis family

### Pupillometry and imaging at baseline

Preoperative overall (OS/OD averaged per patient) QP values were: NPi 3.81 ± 0.69, size before constriction 3.68 ± 0.96 mm, constriction velocity 2.40 ± 1.37 mm/s, and dilation velocity 0.95 ± 0.45 mm/s. Eye‑specific distributions confirmed the presence of pathological NPi values (< 3) in a subset (Right NPi median 3.8 [IQR 0.4; range 2.1–4.8]; Left 3.8 [1.0; 2.5–4.8]). Imaging supported hydrocephalus: Evans index median 0.30 (IQR 0.10; 0.30–0.40), callosal angle 88.9° (IQR 22.7°; 59.8–126.6°), bicaudate index 0.20 (IQR 0.10; 0.20–0.30), third ventricle volume 75.4 cm³ (IQR 37.1; 26.9–239.6), temporal horn diameter 80.0 mm (IQR 21.0; 41.0–102.0), third ventricle width 126.0 mm (IQR 20.0; 66.0–184.0), and periventricular lucency present in essentially all (median 1.0 [IQR 0.0]). Postoperatively, overall QP showed small average increases (NPi 4.03 ± 0.66, pupil size 3.89 ± 0.97 mm, constriction velocity 2.55 ± 1.05 mm/s, dilation velocity 0.98 ± 0.39 mm/s).

### Cross‑sectional associations with radiological severity

Preoperative overall NPi showed a small, non‑significant association with HSS (ρ = 0.37, *n* = 17, *p* = 0.141, q = 0.423) (Table 4). Constriction and dilation velocities behaved similarly (ρ = 0.31, *p* = 0.236; ρ = 0.27, *p* = 0.317; both *n* = 16; q = 0.423). Associations with Evans index alone were negligible for overall NPi (ρ = 0.09, *n* = 17, *p* = 0.735, q = 0.735). After false‑discovery‑rate (FDR) control, no cross‑sectional QP–imaging pair was significant (Tables 2, 3 and 4).

**Table 2:** Spearman correlations — preoperative overall QP vs. imaging

QP metric	Imaging metric	*n*	rho	*p*
NPi	Evans index	17	0.089	0.735
NPi	Third ventricle volume (cm³)	17	0.209	0.421
NPi	Callosal angle (°)	17	−0.552	0.022
NPi	Temporal horn diameter (mm)	17	0.378	0.134
NPi	Bicaudate index	17	0.371	0.143
NPi	Third ventricle width (mm)	17	−0.065	0.805
Constriction velocity (mm/s)	Evans index	16	0.264	0.322
Constriction velocity (mm/s)	Third ventricle volume (cm³)	16	0.130	0.632
Constriction velocity (mm/s)	Callosal angle (°)	16	−0.444	0.085
Constriction velocity (mm/s)	Temporal horn diameter (mm)	16	0.157	0.561
Constriction velocity (mm/s)	Bicaudate index	16	0.289	0.278
Constriction velocity (mm/s)	Third ventricle width (mm)	16	−0.033	0.903
Dilation velocity (mm/s)	Evans index	16	0.323	0.222
Dilation velocity (mm/s)	Third ventricle volume (cm³)	16	0.139	0.608
Dilation velocity (mm/s)	Callosal angle (°)	16	−0.282	0.290
Dilation velocity (mm/s)	Temporal horn diameter (mm)	16	0.129	0.633
Dilation velocity (mm/s)	Bicaudate index	16	0.336	0.203
Dilation velocity (mm/s)	Third ventricle width (mm)	16	−0.095	0.725

Spearman rank correlations (ρ) between preoperative overall quantitative pupillometry metrics (overall NPi, constriction velocity, dilation velocity) and preoperative imaging markers on cranial CT. n indicates the number of patients contributing to each correlation. Imaging markers: EI, ventricular volume (cm³), CA (degrees), THD (mm), BCI, and TVW (mm). Note: p values are two-sided and unadjusted (nominal). Where referenced in the manuscript, q values denote Benjamini–Hochberg false discovery rate (FDR)-adjusted p values within the corresponding analysis family

**Table 3:** Spearman correlations — preoperative overall QP vs. GCS

QP metric	*n*	rho	*p*
NPi	17	−0.007	0.978
Constriction velocity (mm/s)	16	−0.235	0.381
Dilation velocity (mm/s)	16	−0.210	0.435

Spearman rank correlations (ρ) between preoperative overall quantitative pupillometry metrics and neurological status represented by the Glasgow Coma Scale (GCS) at the time of preoperative pupillometry. n indicates the number of patients contributing to each correlation. Note: p values are two-sided and unadjusted (nominal). Where referenced in the manuscript, q values denote Benjamini–Hochberg false discovery rate (FDR)-adjusted p values within the corresponding analysis family

**Table 4:** Spearman correlations — preoperative overall QP vs. composite HSS

QP metric	*n*	rho	*p*
NPi	17	0.372	0.141
Constriction velocity (mm/s)	16	0.314	0.236
Dilation velocity (mm/s)	16	0.267	0.317

Spearman rank correlations (ρ) between preoperative overall quantitative pupillometry metrics and the composite Hydrocephalus Severity Score (HSS; higher = worse). HSS was calculated as the mean of oriented, z‑standardized imaging features (EI, BCI, ventricular volume, THD, TVW, PVL [1/0], and −CA), with a requirement of ≥4/7 components. Note: p values are two-sided and unadjusted (nominal). Where referenced in the manuscript, q values denote Benjamini–Hochberg false discovery rate (FDR)-adjusted p values within the corresponding analysis family

### Associations with neurological status and symptoms

Correlations between preoperative overall QP and GCS were not significant (e.g., overall NPi vs. GCS ρ = −0.01, *n* = 17, *p* = 0.978) (Table 3). Point‑biserial correlations between QP metrics and headache or nausea/vomiting were likewise non‑significant after FDR correction (Table 6).

### Asymmetry analysis

We quantified inter‑eye asymmetry as |OD–OS| for each QP metric. Asymmetry in constriction velocity was significantly associated with hydrocephalus severity: ρ = 0.66, *n* = 16, *p* = 0.0057, q = 0.017 (vs. HSS), as shown in Fig. 2. Other asymmetry measures did not reach significance after FDR control (e.g., NPi asymmetry vs. HSS ρ = −0.35, *n* = 17, *p* = 0.166, q = 0.248). Asymmetry in constriction velocity showed a trend with Evans index (ρ = 0.49, *n* = 16, *p* = 0.054, q = 0.161). These data suggest that inter‑eye disparity in pupillary constriction dynamics captures physiologic severity not fully explained by ventricle size alone (Table 7).

### Adjusted odds of pathological NPi

In logistic models adjusted for age and sex, the odds of pathological overall NPi < 3 were not significantly associated with HSS (odds ratio [OR] per 1‑SD increase 1.04, 95% CI 0.24–4.50, *p* = 0.961, q = 1.000), nor with individual imaging metrics (all q ≥ 0.86; e.g., third ventricle width OR 3.59 [0.17–76.77], *p* = 0.413; Evans index OR 1.44 [0.34–6.14], *p* = 0.621). The adjusted odds ratios for each imaging predictor are shown in Fig. 3. The bicaudate‑index model was not estimable due to limited variability (Table 5).

**Table 5:** Adjusted odds of pathological overall NPi (< 3) — HSS and imaging (per 1 SD, adjusted for age/sex)

Predictor	*n*	OR	95% CI	*p*
HSS (per SD, higher=worse)	17	1.037	[0.24,4.50]	0.961
Evans index (per SD; oriented worse)	17	1.442	[0.34,6.14]	0.621
Third ventricle volume (cm³) (per SD; oriented worse)	17	0.414	[0.04,3.89]	0.440
Callosal angle (°) (per SD; oriented worse)	17	0.364	[0.04,3.08]	0.354
Temporal horn diameter (mm) (per SD; oriented worse)	17	1.689	[0.38,7.57]	0.493
Third ventricle width (mm) (per SD; oriented worse)	17	3.589	[0.17,76.77]	0.413

Age‑ and sex‑adjusted logistic regression models for baseline pathological overall NPi (<3). Predictors are scaled per 1 SD increase; callosal angle is entered with inverted sign so that higher values indicate worse hydrocephalus severity. OR indicates the odds ratio per 1 SD; CI indicates 95% confidence interval. Note: p values are two-sided and unadjusted (nominal). Where referenced in the manuscript, q values denote Benjamini–Hochberg false discovery rate (FDR)-adjusted p values within the corresponding analysis family

### Pre–post change and responder analysis

**Fig. 1:**
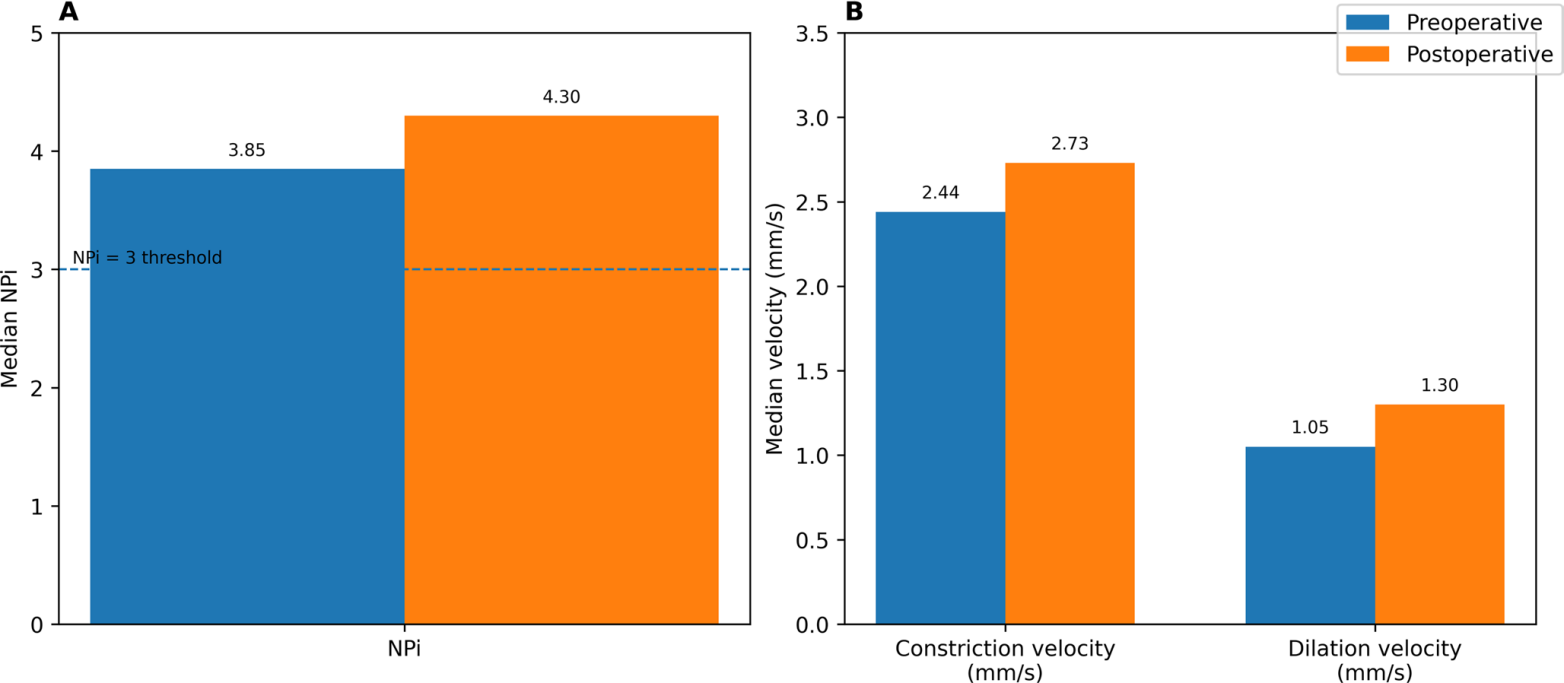
Median pupillometry measures before and after ventriculoperitoneal (VP) shunt placement. (**A**) Median overall Neurological Pupil Index (NPi) preoperatively and on postoperative day 1. The dashed line indicates the pathological threshold (NPi = 3). (**B**) Median overall constriction velocity and dilation velocity (mm/s) preoperatively and on postoperative day 1

Paired overall NPi values were available in 14/17 patients. The median NPi increased from 3.85 preoperatively to 4.30 postoperatively (median Δ + 0.13; Wilcoxon *p* = 0.141). Using a clinically oriented responder definition (ΔNPi ≥ + 0.5), 5/14 (35.7%) patients improved (exact 95% CI 13.0–64.9%). Among those with preoperative NPi < 3, 0/1 converted to NPi ≥ 3 (95% CI 0.0–97.5%). These pre- and postoperative pupillometry trends are visualized in Fig. 1.

## Discussion

This feasibility study sought to determine whether absolute quantitative pupillometry measurements could serve as a bedside indicator of hydrocephalus severity and to assess the impact of ventriculoperitoneal shunting on these values. According to our preliminary findings static pupillary metrics—including the NPi, average constriction velocity and dilation velocity—showed no significant correlation with the radiological HSS or with clinical vigilance. This lack of association aligns with the findings of Campos et al., who reported no correlation between mean NPi and a CT-based hydrocephalus score in a larger prospective aSAH cohort [2]. Taken together, these results suggest that moderate ventricular enlargement may not sufficiently disturb the parasympathetic pupillary reflex arc to cause a reduction in NPi, especially when brainstem pressure remains compensated.

**Fig. 2:**
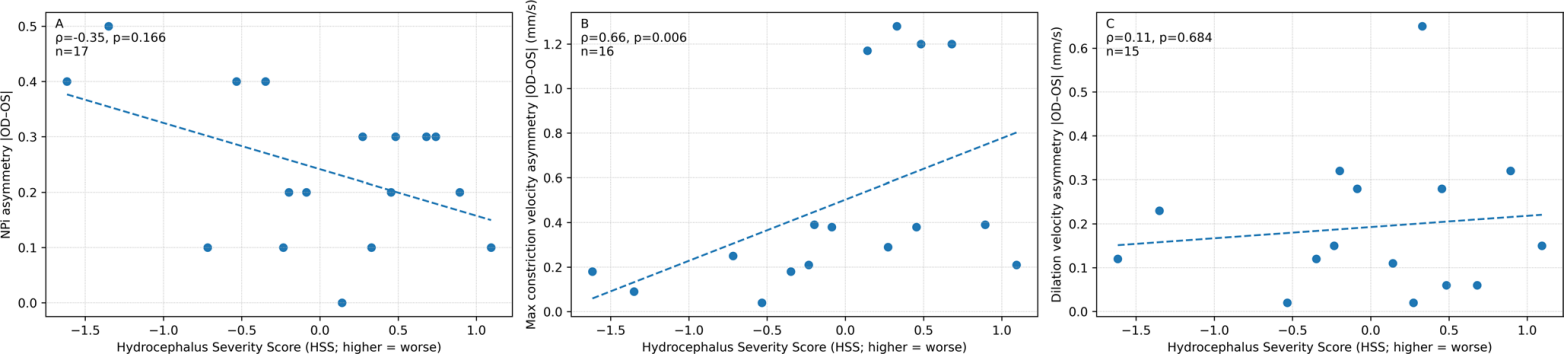
Inter-eye asymmetry in pupillometry metrics versus radiologic hydrocephalus severity. Scatter plots show the relationship between the Hydrocephalus Severity Score (HSS; higher = worse) and inter-eye asymmetry (|OD–OS|) in (**A**) NPi (dimensionless), (**B**) constriction velocity (mm/s), and (**C**) dilation velocity (mm/s). Each point represents one patient. Spearman’s ρ, two-sided p-values, and sample size (n) are displayed in each panel. The dashed line is shown for visualization only

In contrast, we observed that the left–right asymmetry in pupillary constriction velocity correlated strongly with the composite HSS (Fig. 2), aligning with prior observations in stroke patients, where midline shift has been shown to correlate with NPi and constriction velocity [9]. Whereas in stroke a unilateral mass lesion directly compresses one side of the midbrain, post-aSAH hydrocephalus may produce asymmetrical ventricular distension—such as differential temporal horn expansion—leading to lateralised stretching of the Edinger-Westphal–oculomotor pathway on one side. Experimental work in traumatic brain injury supports this interpretation: high-dose propofol and opioid combinations reduce pupillary size and percentage constriction but have a much smaller effect on NPi [7]. Because the NPi algorithm adjusts for baseline pupil size and latency, it may remain stable despite moderate sedation, whereas velocity-based asymmetry can reveal subtle changes, side-to-side differences in parasympathetic efferent conduction. Despite variability in sedation levels in this cohort, the observed pupillary asymmetry persisted, supporting a physiological association between lateralized ventricular pressure and afferent–efferent pathways of the pupillary light reflex.

**Fig. 3:**
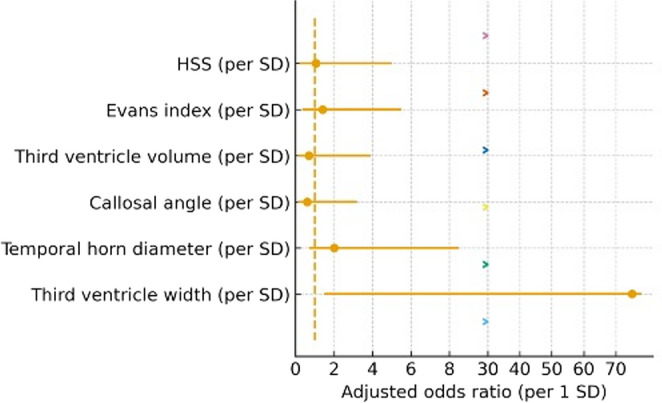
Imaging/HSS predictors for pathological overall NPi (< 3): This forest plot shows the adjusted odds ratios (ORs) and 95 % confidence intervals for each imaging‑derived index (per 1 SD) when predicting a pathological NPi (<3)

A notable finding was that the median post-shunt change in NPi was modest (+ 0.13), but more than one-third of patients experienced a clinically meaningful improvement (ΔNPi ≥ + 0.5). This pattern is illustrated in Fig. 1, highlighting that despite small median changes, several individuals showed clinically relevant improvement. This aspect underscores the importance of monitoring individual NPi trends rather than relying on absolute thresholds. This logistic regression analysis, adjusted for age and sex, implies that none of the individual CT markers or the composite HSS predicted pathological NPi values (< 3); odds ratios were close to unity and confidence intervals crossed 1.0 (Fig. 3). Dynamic changes in NPi have previously been shown to predict early failure of external ventricular drain clamp trials with high specificity [9]. This supports the notion that downward shifts, recovery patterns, and asymmetries in NPi provide more clinically relevant information than static single values. Similarly, in large cohorts of acute brain injury, early NPi declines have been linked to impending transtentorial herniation and worse outcomes, whereas absolute NPi values may remain within normal limits until late in the course [12]. Noteworthily, CT-based indices alone could not predict pathological NPi values (< 3), emphasizing that ventricular size and intracranial pressure are not linearly reflected in pupillary reactivity. Recent studies further support this observation, showing that neither ventricular size on CT nor instantaneous ICP values reliably predict whether a patient will exhibit a pathological NPi (< 3).

In neurocritical care—such as in aSAH with hydrocephalus—quantitative pupillometry has emerged as a valuable monitoring tool, but as a complement rather than a replacement for other modalities. An abnormal NPi can signal critical neurological deterioration, often preceding overt ICP crises [13]. However, data consistently show that ICP and pupillary reactivity are not linearly coupled. Many patients maintain normal NPi values despite elevated ICP, while others develop pupillary abnormalities even at only moderate ICP elevations. Therefore, best practice lies in multimodal monitoring approach that integrates imaging, ICP measurements, absolute pupillometry, and asymmetry trends, enabling clinicians to detect discrepancies and capture critical changes more promptly and reliably than with any single method. Of note, there is the notion that sedation might be a confounder in the findings of QP. However, evidence suggests that commonly used sedatives and opioids have minimal impact on NPi. In a recent prospective cardiac arrest study, propofol and fentanyl sedatives did not significantly alter NPi values, while deep sedation preferentially reduced quantitative PLR [14]. Similarly, in a cohort of severe traumatic brain injury patients, NPi was less affected by sedatives and opioids than were pupil size or percentage constriction [7]. These results are consistent with the present observations: although sedation varied across our patients, the strong correlation between constriction asymmetry and hydrocephalus severity suggests that the effect of sedatives on NPi was minimal in this context. Nevertheless, future studies should standardise sedation regimens and synchronise pupillometry with imaging to minimise potential confounding (Tables 6 and 7).

**Table 6:** Point‑biserial correlations — preoperative overall QP vs. symptoms

Symptom	QP metric	*n*	n_yes	n_no	*r*	*p*
Headache	NPi	17	0	0	0.411	0.101
Headache	Constriction velocity (mm/s)	16	0	0	0.450	0.080
Headache	Dilation velocity (mm/s)	16	0	0	0.588	0.017
Nausea/Vomiting	NPi	17	0	0	−0.037	0.887
Nausea/Vomiting	Constriction velocity (mm/s)	16	0	0	0.257	0.337
Nausea/Vomiting	Dilation velocity (mm/s)	16	0	0	0.193	0.474

Point‑biserial correlations (r) between baseline symptoms (headache; nausea/vomiting) and preoperative overall quantitative pupillometry metrics. The point‑biserial correlation is equivalent to the Pearson correlation between a binary symptom (1 = present, 0 = absent) and a continuous metric. n indicates the number of patients contributing to each correlation; n_yes and n_no show the number with and without the symptom within that analytic sample. Note: p values are two-sided and unadjusted (nominal). Where referenced in the manuscript, q values denote Benjamini–Hochberg false discovery rate (FDR)-adjusted p values within the corresponding analysis family

**Table 7:** Spearman correlations — left–right asymmetry (|OD–OS|) vs. HSS and Evans index

Asymmetry metric	Outcome	*n*	rho	*p*
NPi	HSS	17	−0.352	0.166
NPi	Evans index	17	−0.097	0.712
Constriction velocity (mm/s)	**HSS**	**16**	**0.657**	**0.006**
Constriction velocity (mm/s)	Evans index	16	0.490	0.054
Dilation velocity (mm/s)	HSS	15	0.115	0.684
Dilation velocity (mm/s)	Evans index	15	−0.183	0.514

Spearman rank correlations (ρ) between preoperative inter‑eye asymmetry (|OD–OS|) in quantitative pupillometry metrics and imaging severity (HSS and Evans index). n indicates the number of patients contributing to each correlation. Note: p values are two-sided and unadjusted (nominal). Where referenced in the manuscript, q values denote Benjamini–Hochberg false discovery rate (FDR)-adjusted p values within the corresponding analysis family

Paired overall NPi measurements were available for 14 patients. The median NPi changed from 3.85 preoperatively to 4.30 postoperatively (median Δ=0.13; Wilcoxon p=0.141). Responders defined by ΔNPi ≥ +0.5 comprised 5/14 (35.7%; exact 95% CI 0.13–0.65). Among those with preoperative NPi<3, conversion to NPi≥3 occurred in 0/1 patients (exact 95% CI 0.00–0.97). Left–right asymmetry in constriction velocity (mm/s) correlated with overall hydrocephalus severity (HSS) (ρ=0.66, n=16, p=0.006)

### Clinical implications and future directions

Clinicians should not rely solely on absolute NPi values to estimate hydrocephalus severity; rather, the inter-eye difference in constriction velocity (|OD–OS|) appears to be a more sensitive marker for subtle ventricular expansion. Monitoring trends in NPi and constriction asymmetry over time may help identify patients at risk of shunt failure or progressive hydrocephalus earlier than imaging alone. Given the minimal influence of sedation on NPi automated pupillometry could be integrated into routine neurocritical care monitoring as a dynamic vital sign. Future multicenter studies should incorporate larger cohorts to validate moderate correlations (ρ ≈ 0.3), include additional pupillometric parameters such as latency and percentage constriction, and evaluate long-term outcomes. Moreover, developing algorithms that automatically alert clinicians to significant increases in pupillary asymmetry could enhance early detection and intervention for ventricular enlargement.

### Limitations

This pilot cohort was small and derived from a single center, which may limit generalizability and statistical power. The proprietary nature of the NPi algorithm prevents dissection of its individual components, potentially obscuring subtle relationships between specific pupillary dynamics and ventricular morphology. An MCID for NPi change has not been formally established in shunt-responsive hydrocephalus; we therefore used a measurement-informed responder threshold. Reliability studies suggest that differences in NPi < 0.5 typically fall within expected inter-device/inter-observer variability, supporting ΔNPi ≥ 0.5 as a pragmatic cut-off for meaningful within-patient change [1, 2]. Finally, we did not systematically record intracranial pressure values, which precluded a comprehensive analysis of the interplay between ICP trajectories and pupillary parameters. Future research addressing these limitations will further clarify the utility of quantitative pupillometry for hydrocephalus management.

## Conclusions

In summary, our data reposition quantitative pupillometry in aSAH hydrocephalus from a static severity gauge to a dynamic, physiology-oriented monitor. Single-timepoint values (NPi, mean constriction/dilation velocities) did not mirror ventricular size or bedside status, underscoring a structure–function gap in malresorptive hydrocephalus. By contrast, inter-eye constriction-velocity asymmetry consistently aligned with composite radiologic burden, a pattern that is mechanistically plausible—anisotropic ventricular and cisternal distortion likely imposes lateralized stress on the parasympathetic pupil pathway, which a simple average cannot capture. These observations underscore the clinical value of monitoring trends and asymmetry rather than relying on absolute thresholds, and they advocate integrating dynamic pupillometry into hydrocephalus management while further research refines mechanistic understanding and clinical cut-offs.

## Supplementary Information

Below is the link to the electronic supplementary material.

Supplementary Material 1 (DOCX 0.98 MB)

## Data Availability

The datasets generated and/or analyzed during the current study are available from the corresponding author on reasonable request.

## Declarations

### Compliance with instructions for authors

We confirm that this manuscript conforms to the Neurocritical Care Instructions for Authors, including formatting, ethical requirements, and reporting standards.

### Originality and concurrent submission

We confirm that this work is original, has not been published elsewhere in whole or in part, and is not under consideration by any other journal.

### Reporting checklist

We adhered to the STROBE reporting guideline for cohort studies. A completed STROBE checklist is provided as a supplementary file with the submission.
