# Supplementary material for: Quantitative pupillometry as a sensitive tool for detecting hydrocephalus-related physiologic burden after aSAH: a prospective feasibility study
Source: Neurol Sci. 2026 Mar 3;47(3):316. doi: 10.1007/s10072-026-08928-2 (PMC12953266; doi:10.1007/s10072-026-08928-2)

# **Supplementary Methods**


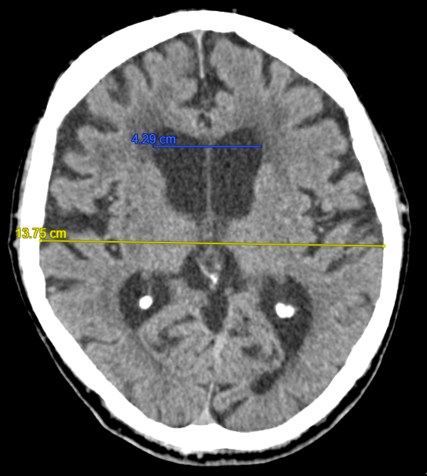


Radiological Parameters

Evans index (EI). Axial slice perpendicular to the AC–PC line at frontal horns; EI = frontal‑horn width / inner‑skull diameter on the same slice. Threshold ≈ 0.30 for ventriculomegaly. (Suppl. Fig. S1A)


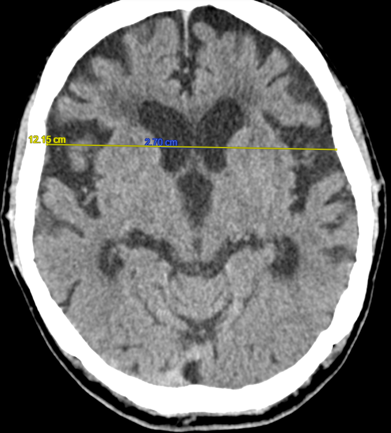


Bicaudate index (BCI). Axial slice at the foramen of Monro; BCI = inter‑caudate distance / inner‑skull diameter at that level. (Suppl. Fig. S1B)


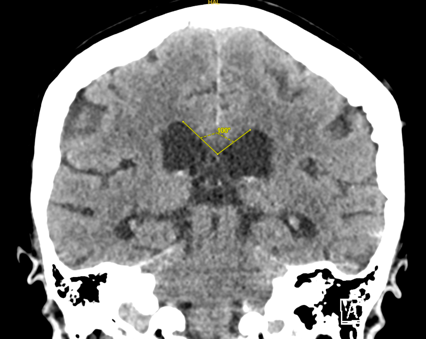


Callosal angle (CA). Coronal image through the posterior commissure, plane orthogonal to AC–PC; angle between medial walls of the frontal horns above the corpus callosum; smaller = worse. (Suppl. Fig. S1C)


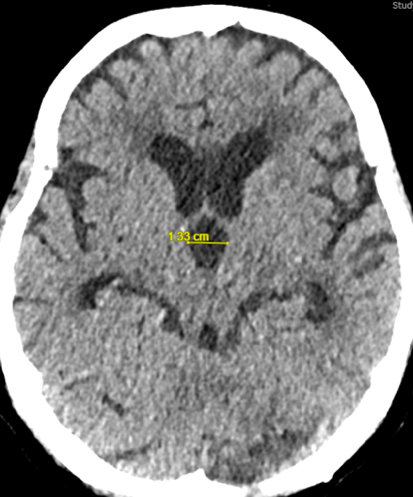
Third‑ventricle width (TVW). Maximal transverse diameter on axial images at the mid‑thalamic level. (Suppl. Fig. S1D)


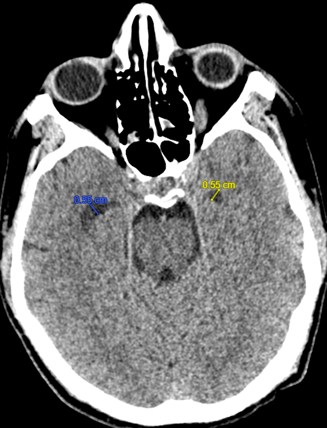


Temporal‑horn diameter (THD). Axial plane at the hippocampal head; measure the widest visible lumen (side‑specific). (Suppl. Fig. S1E)

Ventricular volume. Semi‑automatic segmentation (Origin®, Brainlab v3.1) with manual correction; 3D volume in cm³. Inter‑rater verification on a random 20% subset (target ICC > 0.90). (Suppl. Fig. S2)


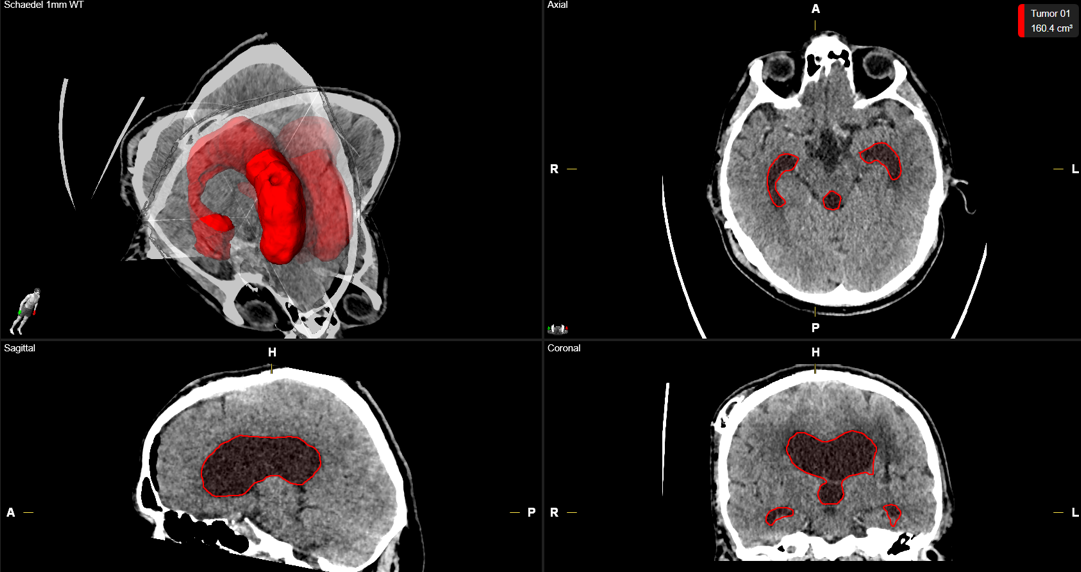

Supplement: Supplementary file 1 — Supplementary Material 1 (DOCX 0.98 MB) [file 10072_2026_8928_MOESM1_ESM.docx]
